# Galectin-3 augments tumor initiating property and tumorigenicity of lung cancer through interaction with β-catenin

**DOI:** 10.18632/oncotarget.3210

**Published:** 2014-12-31

**Authors:** Ling-Yen Chung, Shye-Jye Tang, Yi-Ching Wu, Guang-Huan Sun, Huan-Yun Liu, Kuang-Hui Sun

**Affiliations:** ^1^ Department of Biotechnology and Laboratory Science in Medicine, and Immunity and Inflammation Research Center, National Yang-Ming University, Taipei, Taiwan, ROC; ^2^ Department of Education and Research, Taipei City Hospital, Taipei, Taiwan, ROC; ^3^ Institute of Marine Biotechnology, National Taiwan Ocean University, Keelung, Taiwan, ROC; ^4^ Division of Urology, Department of Surgery, Tri-Service General Hospital and National Defense Medical Center, Taipei, Taiwan, ROC; ^5^ Division of Urology Surgery, Department of Surgery, Taoyuan Armed Forces General Hospital, Taoyuan, Taiwan, ROC

**Keywords:** Cancer stem cells (CSCs), Galectin-3, β-catenin, lung cancer

## Abstract

Cancer stem cells (CSCs) are comprised of a rare sub-population of cells in tumors that have been proposed to be responsible for high recurrence rates and resistance to chemotherapy. Galectins are highly expressed in cancers that correlate with the aggressiveness of tumors. Galectins may also promote the resistance of cancer cells to chemotherapy. However, the role of galectins in CSCs remains unknown. In this study, sphere formation was used to enrich H1299 human lung CSCs that had self-renewal ability, advanced tumorigenic potential, and that highly expressed stem/progenitor cell markers such as Oct4, Sox2, Nanog, and CD133. A novel candidate molecule, galectin-3, for stemness was found in lung CSCs. The expression of galectin-3 robustly increased in lung cancer spheres over serial passages, but its suppression in the H1299 monolayer or spheres resulted in reduced expression of stemness-related genes, sphere-forming ability, tumorigenicity, chemoresistance, and tumor initiation in mice. Notably, the overexpression of galectin-3 in A549 lung cancer cells, which have low capability to grow as tumor spheres, promoted CSC formation. β-catenin activity was increased in H1299 spheres and counteracted by galectin-3 suppression. Thus, galectin-3 may act as a cofactor by interacting with β-catenin to augment the transcriptional activities of stemness-related genes. Furthermore, galectin-3 expression correlated with tumor progression and expressions of β-catenin and CSC marker CD133 in lung cancer tissues. Targeting galectin-3 signaling may provide a new strategy for lung cancer treatment by inhibiting stem-like properties.

## INTRODUCTION

Non-small cell lung cancer (NSCLC) is the leading cause of cancer death worldwide [1]. Combination cytotoxic chemotherapy is preferable as first-line treatment but it has many limitations that are worth noting. More than 50% of patients fail due to an intrinsic resistance or the development of an acquired resistance which develops during the treatment period [2, 3]. In recent years, cancer stem cells (CSCs) have been indicated as key tumor-initiating cells that may play an integral role in recurrence following chemotherapy [4]. Understanding the crucial pathways involved in regulating the development of CSC populations is important when developing novel therapeutic approaches to overcome such drug resistance.

Defined as a rare sub-population of cancer cells with a self-renewal capacity, CSCs have the potential to develop into multiple lineages in tumors and the proliferative ability to continuously expand the population of malignant cells [5]. They have also been identified in some cancers to be critical in sustaining tumor progression and metastasis [6]. Several reports suggest that human lung cancers may also harbor the CSC population [7, 8]. However, a lack of reliable lung stem cell markers has impeded an accurate identification of lung CSCs [9].

The galectin family has high affinity to β-galactoside and shares a conserved carbohydrate recognition domain (CRD) [10]. Galectins are secreted from cells and interact with appropriately glycosylated proteins at the cell surface or within the extracellular matrix to mediate cell-cell or cell-matrix interactions in a glycan-dependent fashion [11]. On the other hand, they can interact with intracellular regulators through protein-protein interactions [12]. Galectins are often highly expressed in cancer cells and cancer-associated stromal cells, and their expression correlates with the aggressiveness of tumors and the acquisition of the metastatic phenotype, indicating that galectins may modulate tumor progression and influence disease outcome [13]. Moreover, galectins may promote cancer cell resistance to platinum-based chemotherapy. Galectin-1 enhances chemoresistance to cisplatin through the MAPK/COX-2 pathway in lung cancer [14]. Galectin-3 promotes chemoresistance in prostate cancer, cholangiocarcinoma, and thyroid carcinoma [15-18], while its inhibition sensitizes prostate cancer cells to cisplatin treatment [16]. Nevertheless, the relationship between galectins and CSCs remains unclear.

This study demonstrated that lung spheres expressed relatively higher levels of galectin-3 over serial passages. Knockdown of galectin-3 in lung adenocarcinomas decreased stemness-related genes like Oct4, Sox2, Nanog, and CD133, as well as the capability of tumor initiation *in vitro* and *in vivo*. The aggressiveness, clonogenicity, and chemoresistance to cisplatin or paclitaxel were also suppressed. Moreover, galectin-3 may act as a cofactor by interacting with β-catenin to augment the transcriptional activity of downstream stemness-related genes. Galectin-3/β-catenin may be a novel mechanism contributing to the initiation and maintenance of lung CSCs, suggesting that targeting galectin-3 may provide an innovative strategy worth considering for lung cancer therapy.

## RESULTS

### Lung tumor spheres exhibited CSC features

**FIGURE 1:**
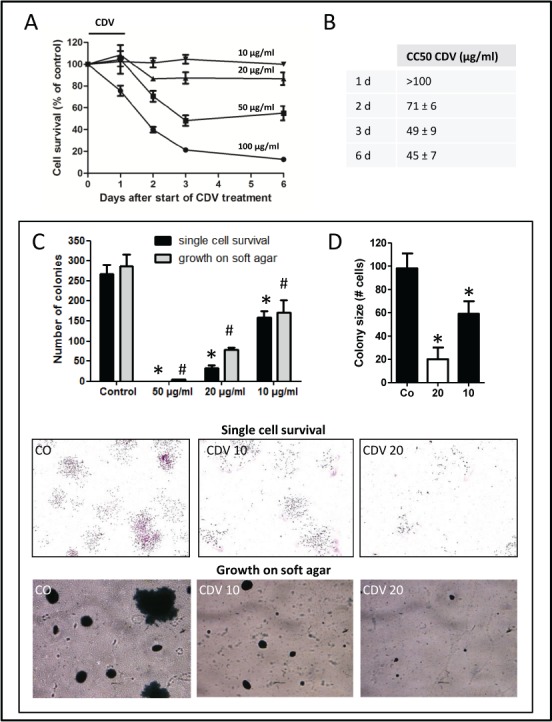
Lung tumor spheres exhibited CSC features (A) The mRNA levels of stemness-related genes in secondary spheres were detected by RT-qPCR and normalized against mRNA levels of parental cells of NSCLC cell lines. (B) The mRNA levels of stemness-related genes in the first and second passages of spheres from H1299 cells were compared with those in monolayer cells. (C) The protein levels of Oct4, Sox2, and Nanog in monolayers (H1299) and sphere (H1299-S) were detected using flow cytometry. (D) Immunofluorescence analysis was performed to detect the expression levels of Oct4, Sox2, Nanog, and CD133 in monolayers (H1299) and spheres (H1299-S). (E) H1299 cells from monolayers (H1299) and spheres (H1299-S) were analyzed for CD44/CD133 double staining by flow cytometry. Control: isotype control. Data represents the mean ± SD of at least 3 independent experiments, *p < 0.05; **p < 0.01.

To enrich the small population of lung CSCs, ten non-small cell lung cancer (NSCLC) cell lines were cultured in defined serum-free selection tumor sphere media. Seven NSCLC cell lines (i.e., H1299, A549, EKVX, H23, Hop62, H522, and H460) had the capacity to propagate in culture as floating spherical colonies. In contrast, three EGFR-mutated cell lines (PC9, H1975, and HCC827) did not grow as lung spheres (data not shown). To assess whether enriched tumor spheres exhibited CSC characteristics, nine stemness-related genes were tested in a panel of seven NSCLC cell line-derived tumor spheres using RT-qPCR (Fig. 1A). The tumor spheres expressed higher mRNA levels of stemness-related markers such as Oct4, Sox2, Nanog, CXCR4, CD133, Smo, and β-catenin than the parental cells (Fig. 1A). Furthermore, H1299-derived tumor spheres expressed much higher levels of these stemness-related genes in the secondary passage compared to monolayers and the first passage (Fig. 1B). It is apparent that increased levels of Oct4, Sox2, Nanog, and CD133 proteins were detected in H1299 spheres compared to those in the parental cells using flow cytometry and immunofluorescence (Figs. 1C and 1D). Over 97% of H1299 spheres were enriched in Oct4^+^, Sox2^+^, or Nanog^+^ cell populations (Fig. 1C). Since CSCs were enriched in CD44^+^ and/or CD133^+^ cell population had been reported [21], H1299 cells from monolayer and tumor spheres were analyzed for CD44/CD133 double-staining by flow cytometry (Fig. 1E). Over 90% of H1299 in both monolayer and sphere cultures were CD44^+^. Only 0.97% of cells in monolayer cultures were CD133^+^, whereas 94.7% of H1299 lung spheres were enriched in CD133^+^ cells. Furthermore, 94.7% of H1299 lung spheres coordinately expressed CD44 and CD133 genes. These results suggested that H1299 spheres were enriched for CSC-like traits.

### Upregulation of galectin-3 in lung CSCs

**FIGURE 2:**
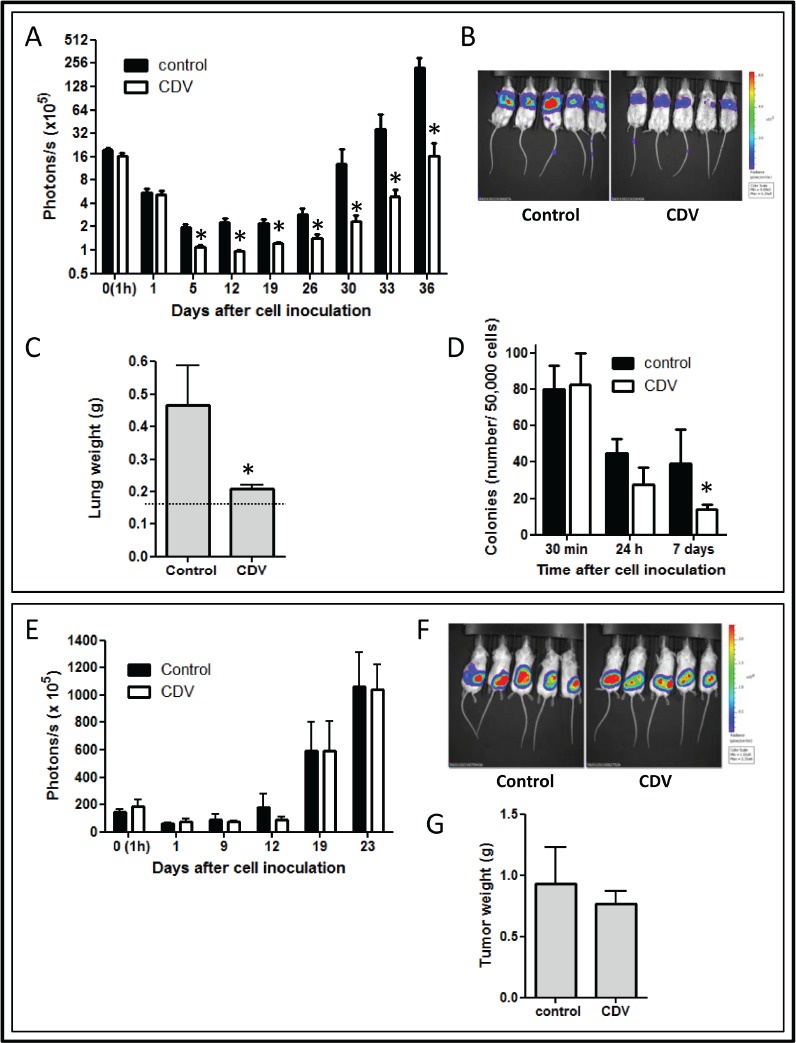
Overexpression of galetin-3 in lung CSCs (A) The mRNA levels of galectins (Gal) in first and secondary passages of H1299 spheres were detected by RT-qPCR and normalized against mRNA levels of monolayers. (B) The mRNA levels of galectin-3 in NSCLC spheres compared to that in monolayers were detected by RT-qPCR. (C) Galectin-3 (Gal-3) was knocked down in H1299 cell line by lentiviral shGal-3 infection. Luciferase shRNA lentivirus (shLuc) was used as a control. The expression levels of galectin-3 were detected using RT-qPCR and Western blotting. (D) The mRNA levels of stemness-associated genes in monolayers (shLuc monolayer), spheres (shLuc sphere), or shGal-3-infected spheres (shGal-3 sphere) were detected by RT-qPCR. (E) shLacZ- or shGal-3-infected H1299 monolayers or spheres were cotransfected with pGL4-Oct4-luc, pGL4-Sox2-luc or pGL4-Nanog-luc, and pRL-SV40. After 48 h, luciferase reporter assays was conducted to investigate Oct4, Sox2, or Nanog promoter activity regulated by galectin-3. (F) The protein levels of Oct4, Sox2, and Nanog in shLuc- or shGal-3-infected H1299 spheres were detected by using flow cytometry. (G) H1299 cells from shLuc- or shGal-3-infected H1299 spheres were analyzed for CD44/CD133 double staining by flow cytometry. Control: isotype control. Data represents the mean ± SD of at least 3 independent experiments, *p < 0.05; **p < 0.01.

The CSCs were considered as the postulated mediators of chemoresistance [22]. Since several reports have indicated that galectins promote the resistance of cancer cells to chemotherapy [14, 18], the expression levels of the galectin family were examined in H1299-derived monolayer and tumor spheres (Fig. 2A). Galectin-3, -4, -7, and -9 were expressed at high levels in H1299 spheres. Galectin-3 was the most robustly expressed in tumor spheres during serial passages (Fig. 2A). As compared to galectin-3 expression, galectin-9 expression was quite low either in H1299 monolayers or tumor spheres (Supplementary Data Fig. 1A). Immunofluorescence staining showed that H1299 spheres were enriched in galectin-3^+^ cells (Supplementary Data Fig. 1B). Moreover, except for H460, most NSCLC-derived tumor spheres had increased galectin-3 (Fig. 2B). Thus, galectin-3 might play important roles for lung tumor initiation and maintenance of CSC properties.

### Suppression of galectin-3 inhibited the self-renewal, chemoresistance, and tumorigenicity of H1299 spheres

To determine the roles of galectin-3 in tumor progression and stemness of lung cancer, lentivirus-mediated delivery of galectin-3 (shGal-3) was used to reduce galectin-3 expression in the H1299 cell line. Compared to cells infected with control virus expressing luciferase shRNA (shLuc), cells infected with shGal-3 virus expressed very low levels of galectin-3 (Fig. 2C). The expression of stemness-related genes was reduced in shGal-3-infected H1299 spheres analyzed by RT-qPCR (Fig. 2D). To examine promoter activities of target genes transactivated by Oct4, Sox2 or Nanog, shLacZ-, or shGal-3-infected H1299 monolayers or spheres were transiently transfected with Oct4, Sox2, or Nanog reporter constructs (Fig. 2E). Enhanced luciferase reporter activities in shLuc-infected spheres were detected compared with those in shLuc-infected monolayer cells, and counteracted by galectin-3 knockdown. Moreover, flow cytometry analysis showed that more than 96% of shLuc-infected spheres were enriched in Oct4^+^, Sox2^+^, or Nanog^+^ cell populations compared with shLuc-infected monolayer, but reduced percentage of Oct4^+^ or Nanog^+^ cells were detected in shGal-3-infected sphere groups (Fig. 2F). The mean fluorescence intensity (MFI) of Oct4, Sox2, and Nanog was increased in shLuc-infected spheres but decreased in shGal-3-infected spheres. The percentage of CD44/CD133 double-positive cell population in shLuc-infected spheres was greater than that in the shGal-3-infected spheres (58.4% vs. 0.06%; Fig. 2G).

**FIGURE 3:**
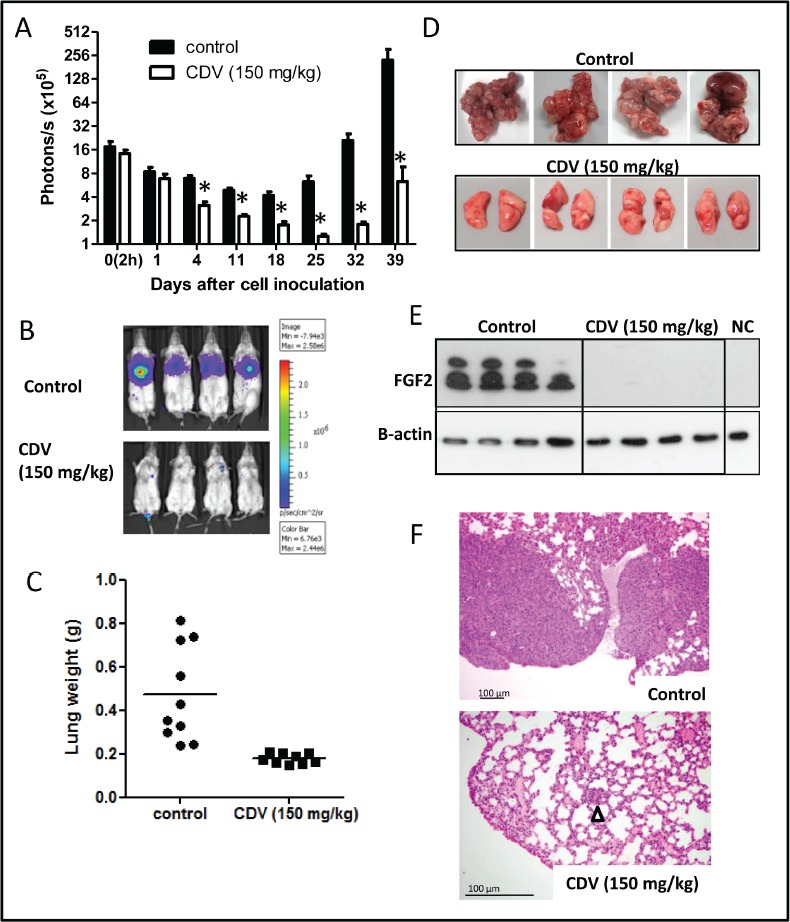
Galectin-3 knockdown in H1299 resulted in reduction of CSC formation, chemoresistance, tumorigenicity, and tumor initiation *in vivo* (A) shLuc- or shGal-3-infected H1299 cells were seeded onto 96-well plates in serum-free tumor sphere media and cultured for 12 days. The primary spheres (1° sphere) were dissociated to single cells and re-seeded to yield the second generation (2° sphere) and third generation of lung spheres (3° sphere). (B) shLuc- or shGal-3-infected parental or sphere cells (500 cells per 6-well plate) were seeded in soft agar for evaluating anchorage-independent tumor growth. (C) To determine the invasive ability, shLuc- or shGal-3-infected spheres (2 × 10^4^ cells) were seeded onto upper side of Matrigel-coated transwell inserts and incubated for 24 h. (D, E) shLuc- or shGal-3-infected monolayers or spheres were cultured in 96-well plates and treated with cisplatin (D) or paclitaxel (E) for 48 h. Cell viability was examined by Celltiter Glo Luminescent Cell Viability Assay. (F) shLuc- or shGal-3-infected spheres were dissociated and inoculated subcutaneously into NOD/SCID mice (10^6^/100 μl cells). Tumor volume was measured every week. Experiments were performed in quadruplicate and the tumor formation was detected 10 weeks after injection. Data represents the mean ± SD of at least 3 independent experiments, *p < 0.05; **p < 0.01.

Since the successful sphere formation of CSCs under serial passages was a key behavior of CSCs for evaluating self-renewal capacity [6], the frequency of tumor sphere generation was analyzed under serial passages. There was a trend of increasing sphere formation frequency within the shLuc-infected H1299 (Fig. 3A). In contrast, the ability of H1299 to form tumor spheres was decreased by shGal-3 knockdown and intensely suppressed after secondary and tertiary passages.

In culture, CSCs were more clonogenic compared to non-CSCs [23]. The shLuc-infected H1299 produced more colonies in soft agar than the shGal-3-infected cells (Fig. 3B). Furthermore, the increased colony-forming capacity in the H1299 spheres under serial passages was restrained by galectin-3 knockdown. The invasive ability was inhibited in shGal-3-infected H1299 spheres (Fig. 3C).

The CSCs reportedly displayed enhanced chemoresistance that might contribute to the survival of CSCs and recurrence of tumors after chemotherapy [24]. When treated with cisplatin or paclitaxel at various concentrations for 48 h, shLuc-infected control cells had a higher IC50 value to cisplatin (IC50: 20.09 ± 0.43 μM) and paclitaxel (IC50: 114.65 ± 8.72 nM) as compared to shGal-3-infected H1299 cells (cisplatin, IC50: 8.80 ± 0.21 μM; paclitaxel, IC50: 39.47 ± 3.23 nM; Figs. 3D and 3E). Furthermore, the H1299 spheres exhibited greater resistance to both cisplatin and paclitaxel than the parental cells. In contrast, the H1299 spheres that carried shGal-3 exhibited increased sensitivity to cisplatin (IC50: 10.34 ± 0.38 μM) and paclitaxel (IC50: 56.81 ± 1.22 nM) as compared to shLuc-infected spheres (cisplatin, IC50: 56.04 ± 2.50 μM; paclitaxel, IC50: 281.95 ± 14.31 nM).

Limiting-dilution transplantation in mouse xenografts is a well-established assay used to characterize CSCs [25]. To assess the tumor-initiating capacity of lung cancer cells, H1299-derived monolayers or tumor spheres were injected subcutaneously in the NOD/SCID mice at limiting-dilution. Mice were injected subcutaneously with either 1 × 10^4^, 1 × 10^5^, or 1 × 10^6^ cells of H1299/shGal-3 monolayers or tumor spheres in the left flank (four mice per group), and the same number of H1299/shLuc control cells in the right flank of the same mice. The H1299-derived sphere cells, but not monolayers or shGal-3-infected sphere cells, generated tumors with cell number as low as 10^4^ cells (Table 1). Furthermore, tumor growth monitored with calipers every week indicated significant decrease in the growth rate of shGal-3-infected H1299 tumors (Fig. 3F). These results revealed that galectin-3 might mediate tumor initiation and tumor growth *in vivo*.

**Table 1:** Tumorigenic potential of shLuc- or shGal-3-infected monolayers or spheres from H1299 cell line

	No. of injected cells	No. of mice with tumor formation/Total No. of mice with cell injection
Monolayers		
H1299/shLuc	1 × 10^4^	0/4
	1 × 10^5^	1/4
	1 × 10^6^	4/4
H1299/shGal-3	1 × 10^4^	0/4
	1 × 10^5^	0/4
	1 × 10^6^	2/4
Spheres		
H1299/shLuc	1 × 10^4^	3/4
	1 × 10^5^	4/4
	1 × 10^6^	4/4
H1299/shGal-3	1 × 10^4^	0/4
	1 × 10^5^	1/4
	1 × 10^6^	3/4

Notes: lentiviral shRNA infected cells were dissociated and subcutaneously injected in NOD/SCID mice (n = 4 per group). The tumor formation was detected 10 weeks after injection.

### Increased galectin-3 interacted with β-catenin in lung CSCs

**FIGURE 4:**
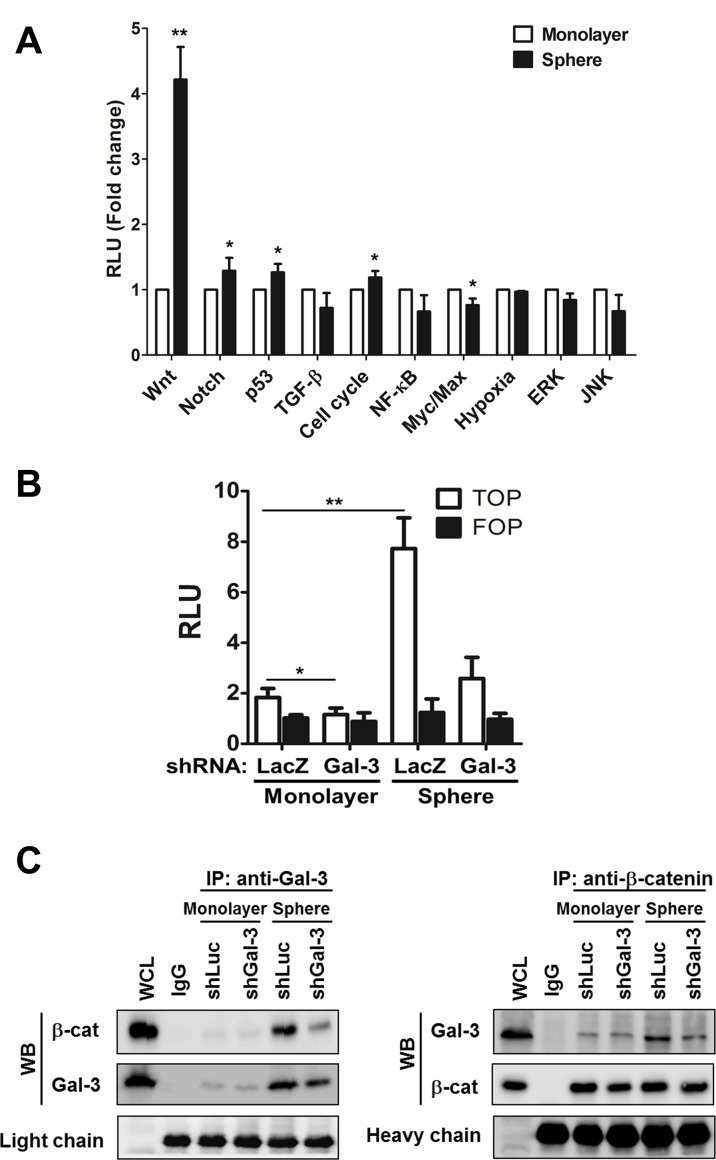
Increased galecitn-3 associated with β-catenin in lung CSCs (A) Cignal Finder Reporter Array consist of 10 dual-luciferase reporter assays was performed. H1299 monolayers or spheres were dissociated and seeded onto 96-well plates (4 × 10^3^ cells). Cells were transfected with the reporter plasmids and incubated for 48 h. Firefly luciferase activity was measured using the Dual-Luciferase Reporter Assay system. (B) The β-catenin promoter activity was examined by TOPFlash system. H1299 monolayers or spheres (4 × 10^3^ cells per 96-well plate) were cotransfected with TOP or FOP reporter construct and pRL-SV40. After 48 h, the firefly luciferase enzyme activity was measured using the Dual-Luciferase Reporter Assay system and normalized to *Renilla* luciferase activity. (C) shLuc- or shGal-3-infected monolayers or spheres were cultured for 3 days and treated with DSS (5 mM) 30 minutes before lysis. We performed immunoprecipitation (IP) in the presence of lactose (5 mM) using anti-galectin-3 (Gal-3) antibody (left) or anti-β-catenin (β-cat) antibody (right), and then immuno-blotted using anti-galectin-3 or anti-β-catenin antibody. IgG, negative control; WCL, whole cell lysate control. Data represents the mean ± SD of at least 3 independent experiments, *p < 0.05; **p < 0.01.

To characterize potential signaling pathways important for lung CSC formation, a signal reporter array that included ten signal transduction pathways that were critical regulators of cancer biology, was performed. There was an elevated expression of the Wnt pathway in H1299 spheres compared to that in the monolayers (Fig. 4A). It was reported that aberrant activation of the Wnt signaling pathway was crucial for self-renewal in CSCs of intestinal, skin, colon, and breast cancers [26, 27]. Therefore, to further identify the relationship between galectin-3 and Wnt signaling in lung CSCs, the TOPFlash/FOPFlash reporter activity was examined in shLuc- or shGal-3-infected monolayers and spheres (Fig. 4B). β-catenin could enter the nucleus to form a transcriptionally active complex with LEF/TCF transcription factors by interacting with coactivators [26]. The TOPFlash reporter construct was specific for β-catenin transcriptional activity, comprising three tandem repeats of TCF binding sites, while FOPFlash was the negative control with a mutated TCF binding site. This study demonstrated that TOPFlash activity was increased in H1299 spheres and counteracted by galectin-3 knockdown (Fig. 4B). Furthermore, galectin-3 directly interacted with β-catenin while galectin-3 knockdown reduced their association in H1299-derived monolayers and spheres using co-immunoprecipitation followed by Western blot (Fig. 4C). Galectin-3 might act as a coactivator by interacting with β-catenin to activate the transcription of stemness-related genes and enhance CSC formation.

### Overexpression of galectin-3 in A549 cells promoted sphere-forming capacity and *in vitro* tumorigenicity

**FIGURE 5:**
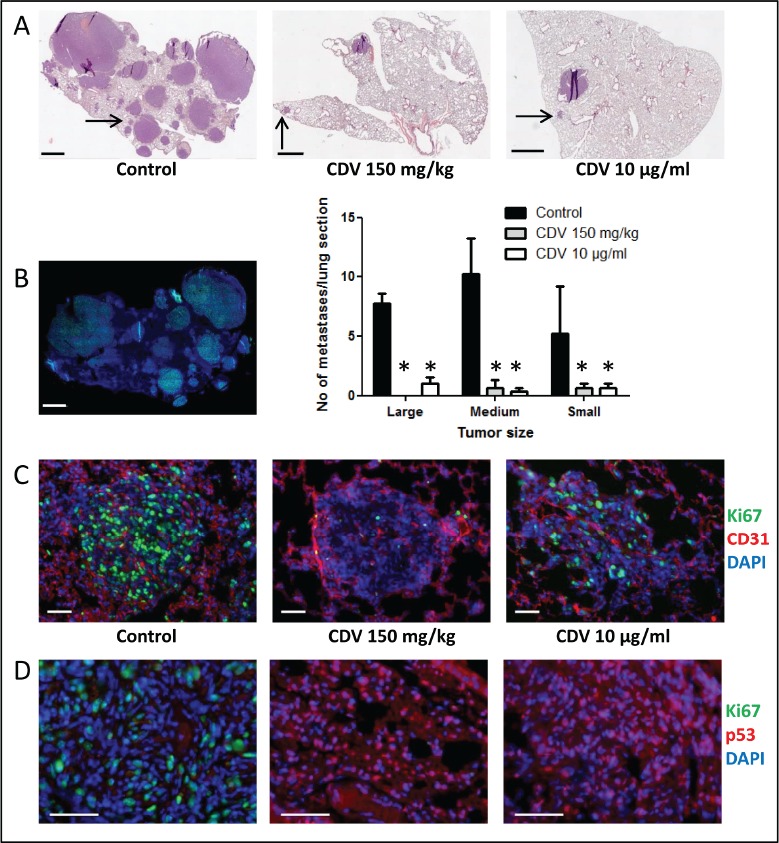
Overexpression of galectin-3 in A549 cells promoted sphere-forming capacity and *in vitro* tumorigenicity (A) The morphology of galectin-3 (Gal-3)- or empty vector (ev)-transduced spheres. The expression levels of galectin-3 in Gal-3- or ev-transduced monolayers or spheres were detected by using RT-qPCR and Western blot. (B) The mRNA levels of stemness-related genes in Gal-3- or ev-transduced A549 monolayers or spheres were detected by RT-qPCR. (C) Gal-3- or ev-transduced A549 monolayers or spheres were cotransfected with pGL4-Oct4-luc, pGL4-Sox2-luc or pGL4-Nanog-luc, and pRL-SV40. After 48 h, luciferase reporter assays were conducted to investigate Oct4, Sox2, or Nanog promoter activity regulated by galectin-3. (D) The protein levels of Oct4, Sox2, or Nanog in Gal-3- or ev-transduced A549 monolayers or spheres were analyzed by flow cytometry. (E) Gal-3- or ev-transduced A549 monolayers or spheres (2 × 10^4^ cells) were seeded onto upper side of Matrigel-coated transwell inserts and incubated for 24 h to determine the invasive ability. (F) Gal-3- or ev-transduced A549 monolayers or spheres (1 × 10^3^ cells per 6-well plate) were seeded in soft agar for evaluating anchorage-independent tumor growth. (G) Gal-3- or ev-transduced A549 were seeded onto 96-well plates in serum-free tumor sphere media and cultured for 30 days. The primary spheres were dissociated to single cells and re-seeded to yield the second generation. Data represents the mean ± SD of at least 3 independent experiments, *p < 0.05; **p < 0.01.

A549 cells that harbor the p53 wild type had decreased capacity to grow as spheres (Fig. 5A; top image). After the A549 cells overexpressed galectin-3 and were cultured in tumor sphere medium, an apparent sub-population grown as tumor spheres was identified (Fig. 5A; top image). Compared to empty vector-transduced A549 monolayers (A549/ev monolayer), the empty vector-transduced spheres (A549/ev sphere) had an increased level of galectin-3, while the galectin-3-overexpressed spheres had a much higher level (A549/Gal-3 sphere; Fig. 5A). To confirm whether these sub-populations were enriched in CSCs, the expression of stemness-related genes were examined in empty vector-transduced monolayers or spheres and galectin-3-overexpressed spheres (Figs. 5B). Galectin-3-overexpressed spheres enriched for cells with increased expressions of Oct4, Sox2, Nanog, and CXCR4 (Fig. 5B). The promoter activities of Oct4, Sox2, and Nanog were also increased in galectin-3-over-expressed spheres (Fig. 5C). Moreover, galectin-3 overexpression increased the proportion of Oct4^+^, Sox2^+^, and Nanog^+^ cells in A549 spheres (Fig. 5D). Galectin-3-overexpressed spheres showed increased invasion, colony formation, and sphere forming ability in the first and secondary passages (Figs. 5E-G).

### Galectin-3 maintained the stemness properties of lung CSCs

**FIGURE 6:**
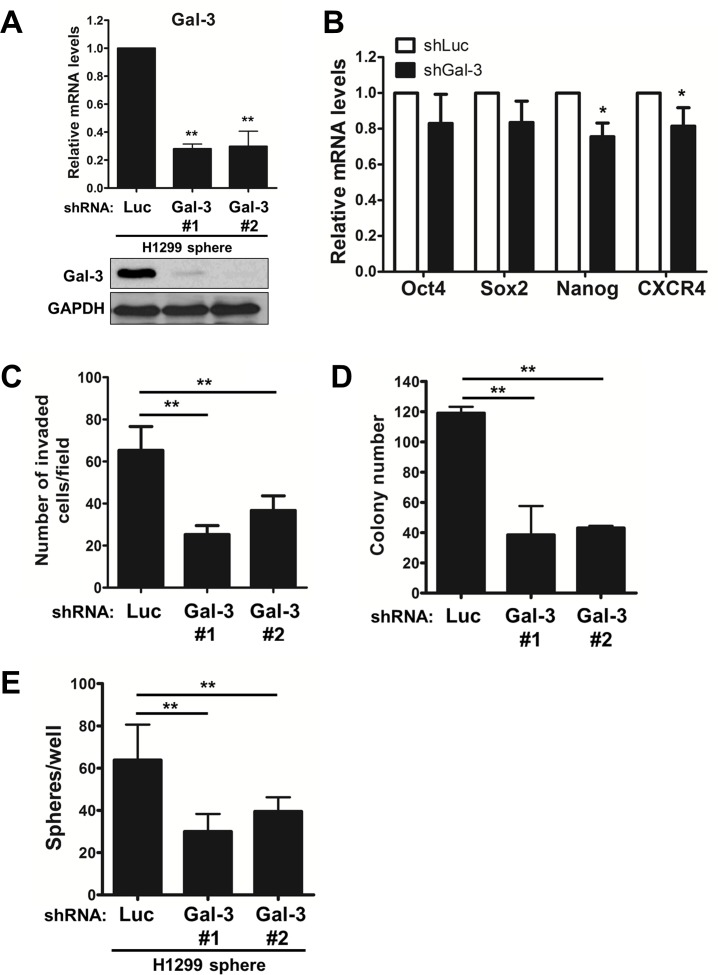
Galectin-3 maintained the stemness properties of lung CSCs Initially, H1299 cells were incubated in tumor sphere medium and grown as spheres. H1299 spheres were then dissociated to single cells and infected with shLuc- or shGal-3 lentivirus. (A) The mRNA levels of galectin-3 were detected by RT-qPCR. (B) The mRNA levels of Oct4, Sox2, Nanog or CXCR4 genes were detected by RT-qPCR. (C) H1299 cells (2 × 10^4^ cells) were analyzed for their invasive ability using Matrigel-coated Transwell assay. (D) After lentivirus infection, H1299 cells (500 cells per 6-well plate) were seeded in soft agar for evaluating anchorage-independent tumor growth. (E) After lentivirus infection, H1299 cells were evaluated the sphere forming ability. Data represents the mean SD of at least 3 independent experiments, *p < 0.05; **p < 0.01.

To demonstrate the effect of galectin-3 on maintaining the stemness properties of lung CSCs, galectin-3 silencing was done in CSC-enriched H1299 spheres. After enrichment of H1299 CSCs, shGal-3 lentivirus was transduced into tumor sphere-generated cells. The mRNA and protein levels of galectin-3 in tumor sphere-generated cells were detected by RT-qPCR and Western Blot (Fig. 6A). The mRNA levels of stemness-related genes, especially Nanog, were reduced in shGal-3-infected H1299 spheres (Fig. 6B). Moreover, the suppression of galectin-3 still had the ability to restrain the invasive ability, colony formation in soft agar, and sphere-forming ability of CSCs-enriched H1299 (Figs. 6C-E).

### Galectin-3 expression correlated with β-catenin, CD133 and tumor progression in lung cancer tissues

**FIGURE 7:**
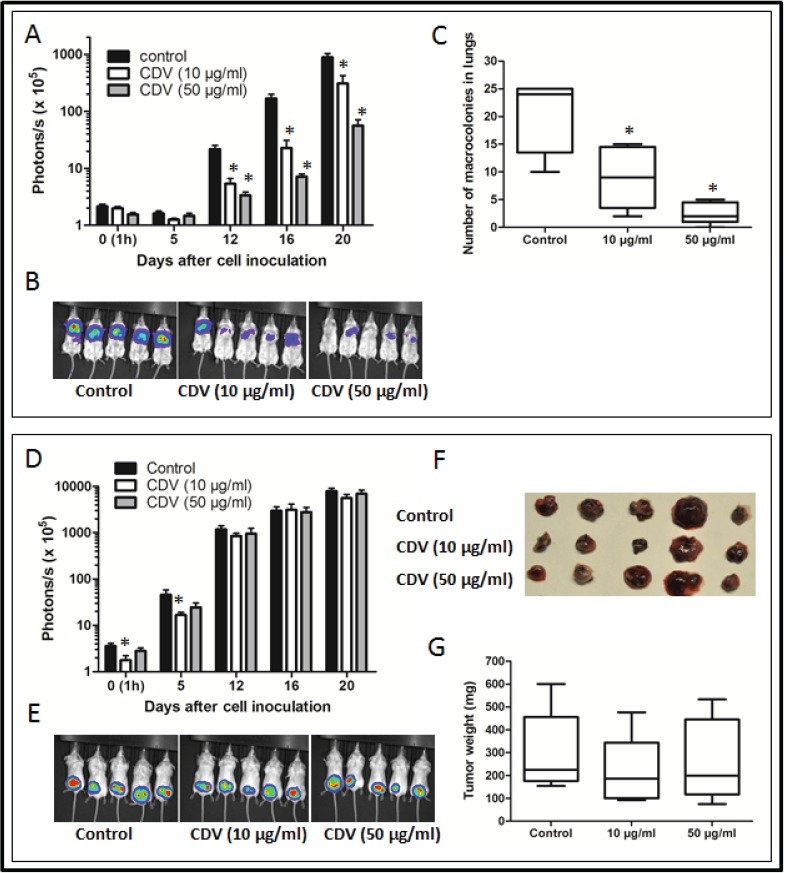
Galectin-3 expression correlated with β-catenin, stemness marker, and tumor progression in tissues of lung cancer (A) IHC staining was performed using anti-galectin-3, anti-β-catenin and anti-CD133 antibodies on tissue microarrays containing samples from 197 patients with NSCLC. (B) Galectin-3 and β-catenin expression levels were verified in well- to poorly-differentiated states of NSCLC. (C) Galectin-3 and β-catenin expression levels were verified in different stages of NSCLC. (D) Statistical analysis of galectin-3 expression was compared with β-catenin in NSCLC tissues. *p < 0.05; **p < 0.01.

To examine the clinical significance of galectin-3 in lung cancer, immunohistochemical staining was conducted on tissue microarrays containing samples from 197 patients with lung cancer, including small cell and non-small cell lung cancer (Fig. 7). Higher levels of both galectin-3 and β-catenin in poorly differentiated and advanced stages of lung cancer were noted (Figs. 7A-C). A positive correlation between galectin-3 and β-catenin was found in lung cancer tissues (Fig. 7D). More importantly, the CD133 positive cells were increased in poorly differentiated lung cancer compared to those in well-differentiated lung cancer (Fig. 7A). There was significant coexpression of galectin-3 and CD133 (Table 2). More specifically, most galectin-3 and β-catenin coexpressed lung tissues (n = 106) expressed CD133 (n = 86; Table 2), suggesting that galectin-3/β-catenin were associated with the stem-like properties of lung cancer.

**Table 2:** Coexpression profiles of CD133, galectin-3 and β-catenin in 197 patients with lung cancer was examined using immunohistochemistry

	Total	Gal-3		*p*-value	Total	Gal-3/β-cat coexpression		*p*-value
	(n = 197)	Low	High		(n = 197)	Low	High	
CD133				< 0.001				< 0.001
Negative	100	56	44		100	80	20	
Positive	97	13	84		97	11	86	

Notes: The expression levels of galectin-3 were categorized as low or high in relation to the median value of the assigned score. The *p*-value based on χ^2^ test statistic was calculated. Gal-3: galectin-3; β-cat: β-catenin.

## DISCUSSION

Galectin-3, a one-CRD galectin belonging to the chimera type, shows pleiotropic biological functions in cell growth, apoptosis induction, tumor progression, and pre-mRNA splicing [28]. Its N-terminal domain is composed of 110-130 amino acids and responsible for multimer formation [11]. The C-terminal CRD contains a NWGR motif that is highly conserved within the BH1 domain of the Bcl-2 family proteins [29]. Galectin-3 can interact with the activated GTP-bound K-Ras and subsequently activate Ras-mediated Akt signaling to inhibit cell apoptosis [30]. Its overexpression has been reported to enhance cell motility and invasiveness *in vitro* of lung cancer cells [31]. Galectin-3 also modulates VEGF- and bFGF-mediated angiogenesis by binding its CRD to integrin αvβ3, and subsequently promoting the growth of new blood vessels [32]. Furthermore, Yang *et al*. suggest that galectin-3 inhibition sensitizes prostate cancer cells to cisplatin treatment through calpain activation [16]. However, the effect of galectin-3 on stemness of lung cancer has not been determined. This study suggests that galectin-3 may act as a cofactor by interacting with β-catenin in the nucleus to enhance the transcriptional activity of stemness-related genes and CSC formation.

Several reports show that galectin-3 is expressed in both cytosol and nucleus, and functions as different mechanisms, depending largely on its subcellular localizations. In nucleus, the tumorigenic potential of galectin-3 may involve the interactions of galectin-3 with β-catenin to enhance the expressions of cyclin D and c-MYC [33] and promote cell cycle progression [28]. Nuclear galectin-3 can regulate gene transcription by enhancing transcription factor association with Sp1 and CRE elements in the gene promoter sequences [28]. On the other hand, cytoplasmic galectin-3 can interact with the activated GTP-bound K-Ras and cause constitutive activation of Ras-dependent PI3K and Raf-1 activation [30]. Galectin-3 can also mediate nuclear β-catenin accumulation by regulating glycogen synthase kinase-3β (GSK-3β) activity in colon cancer [34]. But the mechanism of the multifaceted action of galectin-3 remains largely unknown.

Galectin-3 overexpression is associated with the increased invasiveness of many types of tumor cells, including neuroendocrine tumor pheochromocytoma, ovarian, melanoma, thyroid, and colorectal cancer cells [28, 35]. This effect of galectin-3 is attributed partly to its interaction with a range of the ECM glycoproteins such as fibronectins, collagen IV, elastin, and laminin [28]. The interaction of galectin-3 with cell surface epidermal growth factor receptor (EGFR) and transforming growth factor-β receptor (TGFβR) is also believed to contribute to the increased invasiveness of tumor cells [36]. In the present case, galectin-3 cross-links cell surface molecules to prevent their endocytosis and to activate their signaling. A higher level of circulating galectin-3 is also found in the bloodstream of cancer patients, particularly those with metastasis [37]. In CSC niches, whether galectin-3 is secreted from CSCs or from peripheral stromal cells may augment the self-renewal ability or tumorigenicity remains unknown. The H1299 spheres secrete eight-fold higher levels of galectin-3 than H1299 monolayers, and are counteracted by shGal-3 knockdown (Supplementary Data Fig. 2A). In addition, H1299 cells were treated with recombinant galectin-3 (5 ng/ml) in tumor sphere medium and cultured for 12 days to investigate whether extracellular galectin-3 could augment sphere formation. We found that extracellular galectin-3 could increase the ability of H1299 to form tumor spheres (data not shown). The hypothesis of the present study is that extracellular galectin-3 may promote sphere formation in coordination with EGF or bFGF signaling in tumor sphere medium containing EGF or bFGF.

Wnt/β-catenin signaling is involved in both stem cells and cancer stem cells maintenance [38]. Once β-catenin enters the nucleus, it interacts with LEF/TCF and cofactors to bind to specific promoter targets. Several stemness-related genes, such as Oct4, Sox2, Nanog, and CD44 [39, 40], are the downstream genes of β-catenin. Moreover, it has been demonstrated that Oct4 may be a direct target of β-catenin/TCF-mediated transcription [41]. In the present study, β-catenin reporter activity is significantly enhanced in H1299 spheres compared to monolayer, but is counteracted by galectin-3 suppression (Fig. 4B). Galectin-3 has been identified as a novel binding partner of the β-catenin/TCF complex to colocalize with β-catenin in the nucleus and induce the transcription activity of β-catenin target genes [42]. Galectin-3 can also mediate Akt phosphorylation, then promote GSK-3β phosphorylation and inhibit its activity. Inactivation of GSK-3β leads to β-catenin accumulation, increases nuclear localization and target gene expression [34]. The other molecular mechanisms for the effects of galectin-3 on β-catenin transcriptional activity are worth exploring in the near future.

The CSCs can escape the toxic effects of chemotherapy through a variety of pathways, including the Myc, Akt/PKB, Wnt/β-catenin, Notch, and hedgehog. One such pathway, the Wnt/β-catenin signaling pathway, exhibits enhanced chemoresistance to cisplatin in hepatic CSCs [43]. Furthermore, in c-kit^+^ ovarian CSCs, chemoresistance to cisplatin and paclitaxel has been demonstrated to be enhanced by β-catenin via upregulation of ABCG2 [44]. In the present study, galectin-3 silencing increases drug sensitivity to cisplatin and paclitaxel by regulating ABCB1 and ABCG2 (Figs. 3C and 3D and Supplementary Data Fig. 2B). It can be supposed that galectin-3 mediates the upregulation of ABC transporter pumps through β-catenin.

Another mechanism of chemoresistance that has been well explored in CSCs is the role of B-cell lymphoma-2 (BCL-2) family [45]. This protein can be expressed and affect chemoresistance, resulting from induction by several signaling pathways required for CSC survival, such as IL-4 [46], Akt/PKB signaling [47], or Aurora-A [48]. Galectin-3 has been demonstrated to form heterodimers with Bcl-2, or may directly interact with the mitochondrial permeability transition pore complex and regulate its opening, preventing the release of cytochrome c [49, 50]. Moreover, galectin-3 can also inhibit cisplatin-induced poly(ADP-ribose) polymerase degradation and apoptosis, without alter Bcl-2, Bcl-XL, or Bax expressions [29]. These studies highlight the significance of galectin-3 in both Bcl-2-related and unrelated apoptosis resistance. Targeting galectin-3 may improve clinical outcomes for lung cancer patients by overcoming chemoresistance.

In conclusion, galectin-3 induces stemness properties and enhances the expression of stemness-related genes by its associating with β-catenin. There is a correlation among the expression of galectin-3/β-catenin, lung cancer progression, and stemness. Galectin-3 may be a potential marker of prognosis and a novel target for lung cancer therapy.

## MATERIALS AND METHODS

### Cell lines

Human lung adenocarcinoma H1299 (ATCC no. CRL-5803) and A549 (ATCC no. CCL-185) cell lines were obtained from Bioresource Collection and Research Center (BCRC, Hsinchu, Taiwan). Human lung adenocarcinoma, including EKVX, NCI-H23, HOP62 and NCI-H522, and large cell carcinoma, NCI-H460, were purchased from the National Cancer Institute (Bethesda, MD). The HEK293T cell line was given by Dr. Jason C. Huang (National Yang-Ming University, Taiwan). The NSCLC cell lines were grown in RPMI-1640 media, while the HEK293T cells were growth in DMEM media supplemented with 10% FBS and 1% penicillin/streptomycin (HyClone Laboratories, Inc., Logan, UT).

### Xenograft model

The NOD/SCID mice (NOD.CB17-Prkdcscid/IcrCrlBltw; 6-8 weeks of age) were purchased from BioLASCO (BioLASCO Taiwan Co., Ltd). The animals were raised under specific pathogen-free conditions in the Animal Center of National Yang-Ming University according to the regulations of the Animal Care Committee of National Yang-Ming University. Tumor cells were injected subcutaneously into NOD/SCID mice (10^4^-10^6^/100 μl cells). Tumor volume was measured with a caliper and calculated as length × width × height (in mm^3^) every week.

### Tissue microarrays and immunohistochemistry

Tissue microarray (TMA) slides were purchased from Biomax (US Biomax, Inc., Rockville, MD). The company provided certified documents that all human tissue samples were collected with informed consents from the donors and their relatives. The detailed clinical-pathologic characteristics of 197 patients with lung cancer are listed in Supplementary Data Table 1. Immunohistochemical staining for galectin-3, β-catenin, and CD133 on TMA was performed as described previously [19]. A digital pathology system for scoring stained cells was performed by Aperio ImageScope (Aperio Technologies, Inc., Vista, CA). A staining index was determined by multiplying the score for staining intensity with the score as the percentage of cells stained positively as previously described [20].

### Sphere formation

For the enrichment of lung CSCs, H1299, A549, HOP62, NCI-H522, and NCI-H460 cells were plated at a low density of 5 cells/μl in the tumor sphere medium consisting of serum-free DMEM/F12 (1:1) medium, N2 supplement, 20 ng/ml human recombinant basic fibroblast growth factor (bFGF), and 20 ng/ml epidermal growth factor (EGF) (GibcoBRL Life Technologies). The NCI-H23 and EKVX cells were cultured in the tumor sphere DMEM/F12 medium containing 20 ng/ml EGF and bFGF growth factors supplemented with B27 (GibcoBRL Life Technologies) instead of N2 supplement. The medium was replaced or supplemented with fresh growth factors every 4 days until the cells started to grow and form floating aggregates.

To yield the second generation, primary tumor spheres were collected and dissociated into single cells by incubation in trypsin-EDTA, followed by re-plating of single cells in serum-free tumor sphere medium.

### Galectin-3 knockdown with shRNA

The HEK293T cells (2.4 × 10^5^/ml) were used to produce lentiviral expressing shRNA viruses against galectin-3 (shGal-3) or luciferase as a negative control (shLuc) by cotransfection of pLKO.1-shGal-1, pCMV-ΔR8.91, and pMD.G vectors (National RNAi core Facility, Academia Sinica, Taipei, Taiwan). H1299 monolayers (5 × 10^4^ cells/ml) were infected with shGal-3 or shLuc in the presence of 8 μg/ml protamine sulfate based on the procedures provided by the National RNAi Core Facility, Academia Sinica (Taipei, Taiwan). To knockdown galectin-3 in CSC-enriched H1299 spheres, H1299-derived tumor spheres were dissociated to single cells and re-seeded in 10% FBS-RPMI medium, followed by infection of shRNA against luciferase or galectin-3.

### Quantitative reverse transcription-PCR (RT-qPCR)

Total cellular RNA was extracted using TRIzol reagent (Invitrogen) and 5 μg of extracted RNA samples reversely transcribed into cDNA according to the manufacturer's protocol (Superscript III, Invitrogen). The RT-qPCR was performed using the SYBR Green Mix containing Thermo-Start DNA polymerase (Bio-Rad, Hercules, CA) according to the manufacturer's instructions, as regards the ABI7700 System (Applied Biosystems, Foster City, CA), with specific primers used (Supplementary Data Table 2).

### Immunoprecipitation and Western blotting

The H1299 monolayers or spheres were cultured for 3 days and treated with a membrane-permeable chemical crosslinker, disuccinimidyl suberate (DSS; Thermo Scientific, Pittsburg, PA, USA) for 30 minutes before lysis. Whole cell lysates were harvested and immunoprecipitated in the presence of lactose (5 mM) by the indicated primary antibodies (1 μg) or non-specific IgG as a negative control, which was conjugated to protein G magnetic beads (20 μl; Millipore) at 4°C overnight. The immunoprecipitates were resolved by 12% SDS-PAGE and then transferred to a PVDF membrane (Millipore). The membrane was blotted with the indicated primary antibodies and then with HRP-conjugated secondary antibodies.

### Flow cytometry

Initially, to examine the protein levels of transcriptional factors Oct4, Sox2, or Nanog in H1299 monolayers or spheres, the cells (5× 10^­­­­5^ cells/ml) were fixed and permeabilized with BD Cytofix/Cytoperm^TM^ Fixation/Permeabilization kit (BD Biosciences) for 15 minutes, and incubated with primary anti-Oct4, anti-Sox2, or anti-Nanog antibodies (Cell Signaling) in BD Perm/Wash^TM^ buffer for 1 hour at 4°C. After washing with Perm/Wash^TM^ buffer, the cells were incubated in the dark with FITC-conjugated secondary antibodies. For CD133/CD44 double staining, the cells were first blocked with blocking reagent for 5 min and coincubated with anti-CD133/1-PE (Miltenyi Biotec) and anti-CD44-FITC (eBioscence) antibodies for 30 min. As a negative control, the cells were stained with the appropriate isotype control. Flow cytometry was performed on a BD FACSCalibur flow cytometer (BD Biosciences).

### Immunofluorescence

The H1299 cells (10^4^ cells/24-well) were seeded onto cover-slips pre-treated with poly-L-Lysine (Sigma-Aldrich) at 37°C overnight. After 12 days, H1299 spheres were formed and fixed in PBS containing 4% para-formaldehyde and 400 mM sucrose, and permeabilized in 1% Triton X-100. After washing, the cells were blocked in 5% BSA and incubated with anti-galectin-3, anti-Oct4, anti-Nanog, or anti-Sox2 primary antibodies at 4°C overnight. The cells were then incubated with FITC-conjugated secondary antibodies for 1 h. For CD133 staining, the cells or spheres were incubated with CD133-PE (Miltenyi Biotec) for 30 min. After counter-staining with DAPI (Sigma-Aldrich) for 10 min, the cells were mounted and analyzed by fluorescence microscopy.

### Colony formation assay

H1299 (500 cells per 6-well) or A549 (1000 cells per 6-well) cells were suspended in 0.33% Bacto agar (Sigma-Aldrich) were layered over 0.5% Bacto agar. After incubation for 12 or 30 days, the cells were fixed and stained with Giemsa stain for calculating the number of colonies.

### Invasion assay

The invasive ability of tumor cells were evaluated by Transwell assay (Costar, 8-μm pore; Corning, NY) as previously described [19].

### Chemo-drug sensitivity

The drug sensitivity of H1299 parental cells and spheres to cisplatin or paclitaxel (Sigma-Aldrich) was measured by Celltiter Glo Luminescent Cell Viability Assay (Promega). Briefly, the cells were seeded onto 96-well plates and cultured in complete medium. After 24 h, the cells were treated with various concentrations of cisplatin or paclitaxel for 48 h. Cell viability was measured according to the manufacturer's instructions (Promega).

### Reporter assay

To pinpoint the signaling pathways involved in galectin-3-mediated stemness-related genes in lung CSCs, Cignal Finder Reporter Array consisting of 10 dual-luciferase reporter assays was performed according to the manufacturer's instructions (Qiagen). To examine the β-catenin transcriptional activity, the TOPFlash system was performed. The TOP reporter construct was specific for β-catenin transcriptional activity, comprising three tandem repeats of TCF binding sites. The FOP reporter was the negative control with a mutated TCF binding site. The H1299 cells were cotransfected with TOP or FOP reporter construct and pRL-SV40. After 48 h, the firefly luciferase enzyme activity was measured using the Dual-Luciferase Reporter Assay system Kit according to the manufacturer's instructions (Promega) and normalized to *Renilla* luciferase activity.

### Statistical analysis

Data were expressed as mean ± S.D. and statistical significance was assessed by ANOVA. For human tissue microarray studies, non-parametric Mann-Whitney U-test was used to test gene expression relevance between different stages and the Pearson's chi-square test was used to test coexpression profiles of CD133, galectin-3 and β-catenin. For dual staining of human tissue microarrays, the Spearman correlation method was used to evaluate the association of scores. Statistical significance was set at *p*<0.05.

## SUPPLEMENTARY MATERIAL FIGURES AND TABLES


