# Supplementary material for: Galectin-3 augments tumor initiating property and tumorigenicity of lung cancer through interaction with β-catenin
Source: Oncotarget. 2014 Dec 31;6(7):4936–52. doi: 10.18632/oncotarget.3210 (PMC4467125; doi:10.18632/oncotarget.3210)
Supplement: Supplementary file 1 [file oncotarget-06-4936-s001.pdf]

Galectin-3 augments tumor initiating property and tumorigenicity of lung cancer through interaction with β-catenin

Supplementary Material

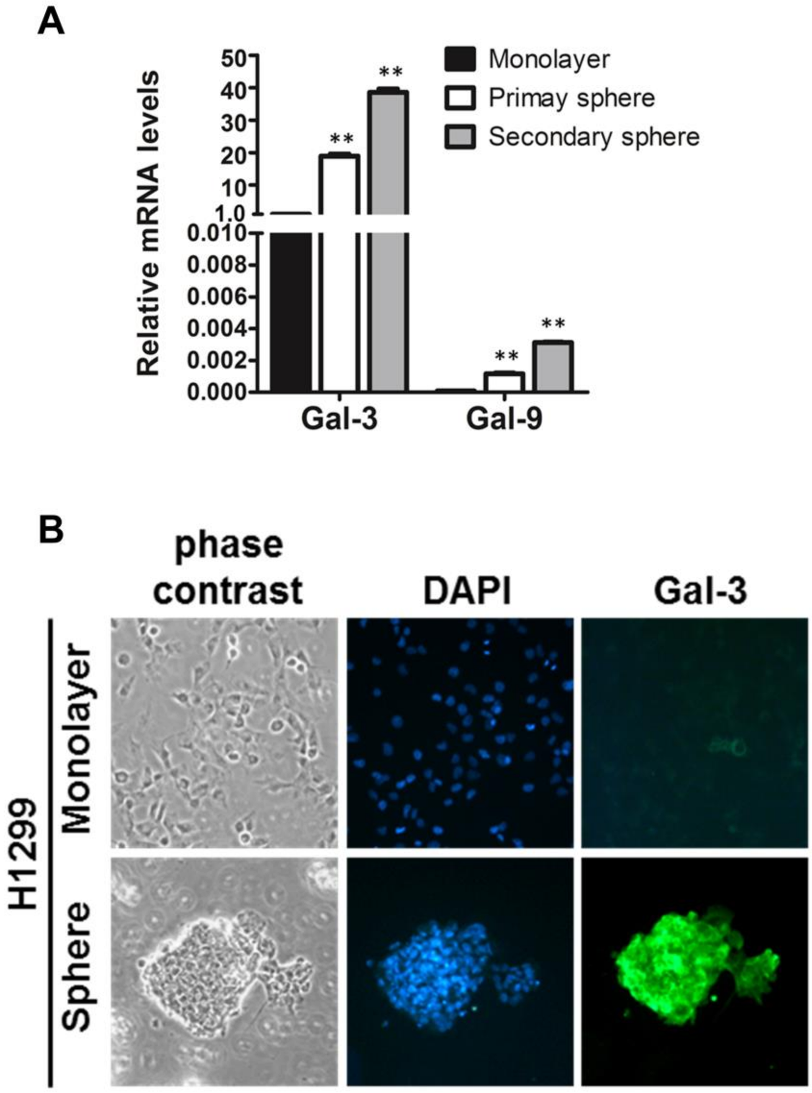

FIGURE S1: Galectin-3 was highly expressed in H1299 spheres. (A) The mRNA level of galectin-3 (Gal-3) and galectin-9 (Gal-9) in the first and second passages of spheres was detected by RT-qPCR. The expression levels of galectin-9 were

normalized with the amount of galectin-3. **(B)** Immunofluorescence analysis was performed to detect the expression levels of galectin-3 (Gal-3) in H1299 monolayers and spheres. Data represents the mean ± SD of at least 3 independent experiments, $*p < 0.05$; $**p < 0.01$.

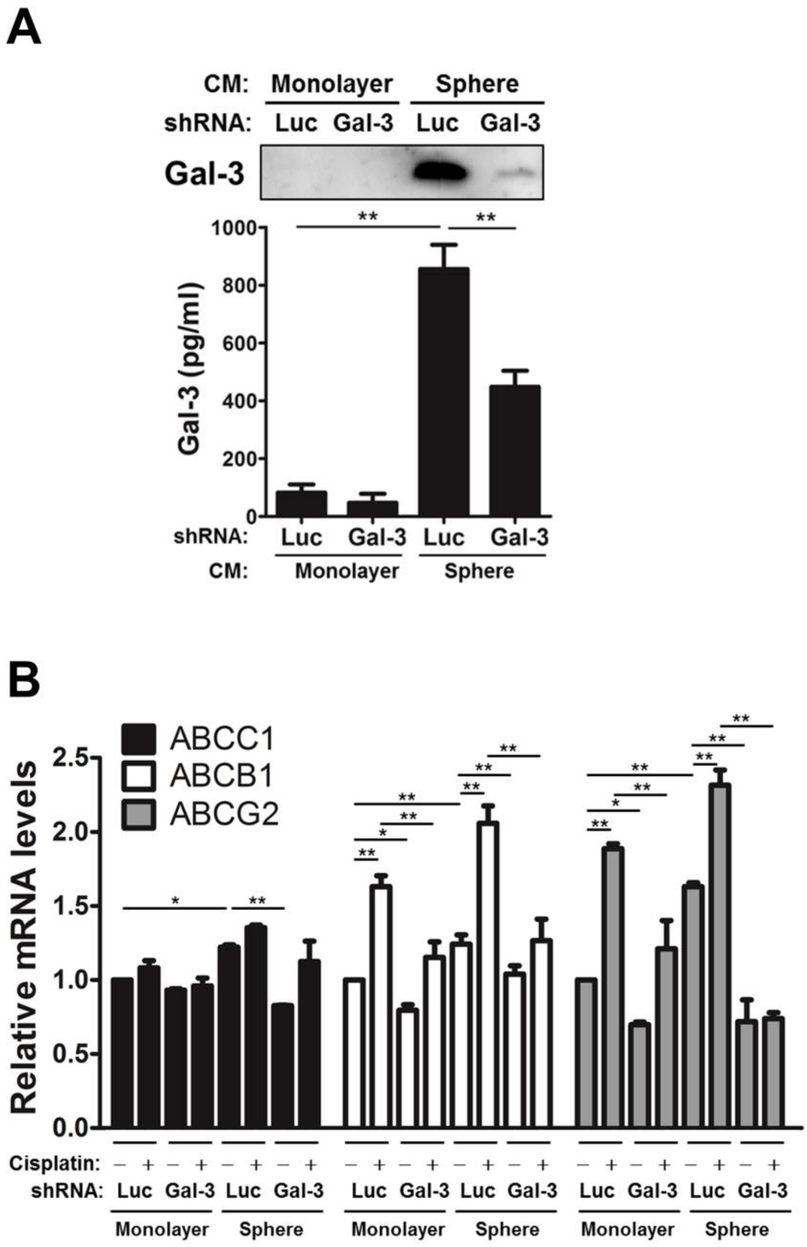

FIGURE S2. Galectin-3 was highly secreted from H1299 cell line and regulated

the expression of drug-resistant genes. (A) The secreted levels of galectin-3 from H1299 monolayers or spheres were detected using direct ELISA and Western blotting. **(B)** The mRNA levels of drug-resistant genes in monolayers or spheres were detected by RT-qPCR. Data represents the mean ± SD of at least 3 independent experiments, *$p < 0.05$; **$p < 0.01$.

Supplementary Data Table 1. Clinicopathologic characteristics of patients with lung cancer included in this study

Case	Age	Sex	Pathology	Grade	Stage	TNM
1	51	M	Squamous cell carcinoma	1	I	T2N0M0
2	60	M	Squamous cell carcinoma	2	I	T2N0M0
3	24	M	Squamous cell carcinoma	2	II	T2N1M0
4	74	M	Squamous cell carcinoma	1	II	T3N0M0
5	68	M	Squamous cell carcinoma	1	I	T2N0M0
6	45	M	Squamous cell carcinoma	1	II	T3N0M0
7	56	M	Squamous cell carcinoma	1	I	T2N0M0
8	66	M	Squamous cell carcinoma	1	I	T2N0M0
9	54	M	Squamous cell carcinoma	1	I	T2N0M0
10	61	M	Squamous cell carcinoma	2	III	T3N1M0
11	39	M	Squamous cell carcinoma	2	II	T2N1M0
12	44	M	Squamous cell carcinoma	1	II	T2N1M0
13	50	M	Squamous cell carcinoma	1	I	T2N0M0
14	70	M	Squamous cell carcinoma	1	I	T2N0M0
15	59	M	Squamous cell carcinoma	2	I	T2N0M0
16	47	M	Squamous cell carcinoma	1	I	T2N0M0
17	58	M	Squamous cell carcinoma	2	II	T2N1M0
18	65	M	Squamous cell carcinoma	2	I	T2N0M0
19	57	M	Squamous cell carcinoma	1	II	T3N0M0
20	56	M	Squamous cell carcinoma	1	I	T2N0M0
21	58	F	Squamous cell carcinoma	1	I	T1N0M0
22	74	M	Squamous cell carcinoma	1	II	T2N1M0
23	61	M	Squamous cell carcinoma	2	II	T2N1M0
24	50	M	Squamous cell carcinoma	2	I	T2N0M0
25	73	M	Squamous cell carcinoma	1	II	T2N1M0
26	76	M	Squamous cell carcinoma	2	I	T2N0M0
27	53	M	Squamous cell carcinoma	2	I	T2N0M0
28	56	M	Squamous cell carcinoma	2	II	T3N0M0
29	65	M	Squamous cell carcinoma	2	II	T3N0M0
30	67	M	Squamous cell carcinoma	3	I	T1N0M0
31	60	M	Squamous cell carcinoma	3	II	T2N1M0
32	56	M	Squamous cell carcinoma	2	II	T3N0M0
33	59	M	Squamous cell carcinoma	-	II	T2N1M0

**34**	53	F	Squamous cell carcinoma	2	II	T2N1M0
**35**	46	F	Squamous cell carcinoma	2	I	T1N0M0
**36**	70	F	Squamous cell carcinoma	2	II	T2N1M0
**37**	68	M	Squamous cell carcinoma	2	II	T2N1M0
**38**	45	M	Squamous cell carcinoma	2	I	T2N0M0
**39**	63	M	Squamous cell carcinoma	2	II	T2N1M0
**40**	60	M	Squamous cell carcinoma	2	I	T2N0M0
**41**	39	F	Squamous cell carcinoma	2	II	T2N1M0
**42**	47	M	Squamous cell carcinoma	2	II	T2N1M0
**43**	54	M	Squamous cell carcinoma	2	III	T2N3M0
**44**	48	M	Squamous cell carcinoma	2	I	T2N0M0
**45**	79	M	Squamous cell carcinoma	2	I	T2N0M0
**46**	50	M	Squamous cell carcinoma	2	III	T3N1M0
**47**	57	M	Squamous cell carcinoma	3	II	T2N1M0
**48**	65	M	Squamous cell carcinoma	3	II	T2N1M0
**49**	48	F	Squamous cell carcinoma	2	I	T2N0M0
**50**	60	M	Squamous cell carcinoma	2	I	T2N0M0
**51**	63	M	Squamous cell carcinoma	2	II	T2N1M0
**52**	49	F	Squamous cell carcinoma	3	II	T2N1M0
**53**	71	M	Squamous cell carcinoma	2	I	T2N0M0
**54**	45	F	Squamous cell carcinoma	2	III	T4N0M0
**55**	61	F	Squamous cell carcinoma	2	I	T2N0M0
**56**	61	M	Squamous cell carcinoma	2	I	T2N0M0
**57**	62	M	Squamous cell carcinoma	2	II	T3N0M0
**58**	71	M	Squamous cell carcinoma	2	I	T2N0M0
**59**	54	F	Squamous cell carcinoma	-	II	T2N1M0
**60**	56	M	Squamous cell carcinoma	-	II	T2N1M0
**61**	64	M	Squamous cell carcinoma	2	I	T2N0M0
**62**	55	M	Squamous cell carcinoma	2	I	T2N0M0
**63**	60	M	Squamous cell carcinoma	2	I	T2N0M0
**64**	52	M	Squamous cell carcinoma	2	III	T4N1M0
**65**	61	F	Squamous cell carcinoma	2	I	T2N0M0
**66**	69	M	Squamous cell carcinoma	2	I	T2N0M0
**67**	62	M	Squamous cell carcinoma	2	I	T2N0M0
**68**	49	M	Squamous cell carcinoma	3	I	T2N0M0
**69**	55	F	Squamous cell carcinoma	3	I	T2N0M0
**70**	61	M	Squamous cell carcinoma	3	I	T2N0M0

**71**	67	M	Squamous cell carcinoma	2	I	T2N0M0
**72**	47	M	Squamous cell carcinoma	1	I	T2N0M0
**73**	72	M	Squamous cell carcinoma	1	I	T2N0M0
**74**	55	M	Squamous cell carcinoma	3	II	T2N1M0
**75**	68	M	Squamous cell carcinoma	3	II	T3N0M0
**76**	65	M	Squamous cell carcinoma	3	I	T2N0M0
**77**	71	M	Squamous cell carcinoma	3	II	T2N1M0
**78**	59	M	Squamous cell carcinoma	3	II	T2N1M0
**79**	43	M	Adenosquamous carcinoma	-	I	T2N0M0
**80**	49	M	Adenosquamous carcinoma	-	I	T2N0M0
**81**	59	M	Adenosquamous carcinoma	-	I	T2N0M0
**82**	70	M	Adenosquamous carcinoma	-	I	T2N0M0
**83**	61	M	Adenosquamous carcinoma	-	I	T2N0M0
**84**	67	F	Adenosquamous carcinoma	-	II	T2N1M0
**85**	70	M	Adenosquamous carcinoma	-	I	T2N0M0
**86**	60	F	Adenosquamous carcinoma	-	I	T1N0M0
**87**	64	M	Adenosquamous carcinoma	-	I	T2N0M0
**88**	62	F	Adenocarcinoma	2	I	T1N0M0
**89**	57	M	Adenocarcinoma	1	I	T2N0M0
**90**	57	F	Adenocarcinoma	1	I	T1N0M0
**91**	60	F	Adenocarcinoma	1	III	T3N1M0
**92**	62	F	Adenocarcinoma	1	I	T2N0M0
**93**	61	M	Adenocarcinoma	1	II	T2N1M0
**94**	66	M	Adenocarcinoma	2	I	T2N0M0
**95**	50	F	Adenocarcinoma	2	I	T2N0M0
**96**	42	F	Adenocarcinoma	2	III	T3N1M0
**97**	55	M	Adenocarcinoma	2	I	T2N0M0
**98**	65	M	Adenocarcinoma	2	I	T2N0M0
**99**	64	F	Adenocarcinoma	2	I	T1N0M0
**100**	50	F	Adenocarcinoma	3	I	T2N0M0
**101**	50	F	Adenocarcinoma	3	I	T2N0M0
**102**	71	M	Adenocarcinoma	3	I	T2N0M0
**103**	63	M	Adenocarcinoma	3	I	T2N0M0
**104**	62	M	Adenocarcinoma	3	I	T2N0M0
**105**	50	M	Adenocarcinoma	2	II	T3N0M0
**106**	68	F	Adenocarcinoma	3	I	T2N0M0
**107**	53	M	Adenocarcinoma	2	I	T2N0M0

**108**	46	M	Adenocarcinoma	2	I	T2N0M0
**109**	59	M	Adenocarcinoma	3	II	T2N1M0
**110**	70	M	Adenocarcinoma	3	I	T2N0M0
**111**	60	F	Adenocarcinoma	3	I	T2N0M0
**112**	70	M	Adenocarcinoma	3	I	T2N0M0
**113**	57	F	Adenocarcinoma	3	I	T2N0M0
**114**	68	M	Adenocarcinoma	2	I	T2N0M0
**115**	59	F	Adenocarcinoma	3	I	T2N0M0
**116**	37	M	Adenocarcinoma	2	I	T2N0M0
**117**	67	F	Adenocarcinoma	2	I	T2N0M0
**118**	57	M	Adenocarcinoma	2	I	T1N0M0
**119**	61	M	Adenocarcinoma	2	I	T2N0M0
**120**	64	F	Adenocarcinoma	2	I	T2N0M0
**121**	70	M	Adenocarcinoma	2	I	T1N0M0
**122**	48	F	Adenocarcinoma	2	I	T2N0M0
**123**	66	F	Adenocarcinoma	2	I	T2N0M0
**124**	52	F	Adenocarcinoma	2	II	T2N1M0
**125**	60	M	Adenocarcinoma	1	I	T2N0M0
**126**	56	M	Adenocarcinoma	3	I	T2N0M0
**127**	54	M	Adenocarcinoma	2	II	T2N1M0
**128**	57	F	Adenocarcinoma	2	I	T2N0M0
**129**	52	F	Adenocarcinoma	2	I	T2N0M0
**130**	70	M	Adenocarcinoma	2	I	T2N0M0
**131**	66	M	Adenocarcinoma	2	I	T1N0M0
**132**	58	M	Adenocarcinoma	2	II	T2N1M0
**133**	70	M	Adenocarcinoma	2	I	T1N0M0
**134**	60	F	Adenocarcinoma	3	I	T2N0M0
**135**	68	M	Adenocarcinoma	3	I	T2N0M0
**136**	61	M	Adenocarcinoma	2	I	T2N0M0
**137**	62	M	Mucinous adenocarcinoma	3	II	T2N1M0
**138**	67	M	Adenocarcinoma	3	I	T2N0M0
**139**	45	M	Adenocarcinoma	2	I	T2N0M0
**140**	53	M	Adenocarcinoma	2	I	T2N0M0
**141**	60	M	Adenocarcinoma	2	I	T2N0M0
**142**	62	M	Adenocarcinoma	3	I	T2N0M0
**143**	62	F	Adenocarcinoma	3	I	T2N0M0
**144**	62	M	Mucinous adenocarcinoma	1	I	T2N0M0

**145**	37	F	Adenocarcinoma	2	I	T2N0M0
**146**	53	M	Adenocarcinoma	2	I	T2N0M0
**147**	48	M	Mucinous adenocarcinoma	2	II	T2N1M0
**148**	59	F	Adenocarcinoma	2	I	T2N0M0
**149**	43	F	Adenocarcinoma	3	III	T2N2M0
**150**	51	M	Adenocarcinoma	3	I	T2N0M0
**151**	50	M	Adenocarcinoma	3	I	T2N0M0
**152**	50	F	Adenocarcinoma	3	II	T3N0M0
**153**	64	M	Adenocarcinoma	3	II	T2N1M0
**154**	62	M	Adenocarcinoma	3	I	T1N0M0
**155**	65	M	Adenosquamous carcinoma	-	I	T2N0M0
**156**	67	F	Adenocarcinoma	2	I	T2N0M0
**157**	51	F	Adenocarcinoma	3	I	T2N0M0
**158**	61	M	Adenocarcinoma	3	I	T2N0M0
**159**	58	F	Adenocarcinoma	3	I	T2N0M0
**160**	49	F	Adenocarcinoma	3	II	T2N1M0
**161**	60	M	Adenocarcinoma	3	I	T1N0M0
**162**	65	M	Adenocarcinoma	3	I	T2N0M0
**163**	48	F	Adenocarcinoma	3	I	T2N0M0
**164**	65	M	Adenocarcinoma	3	I	T2N0M0
**165**	63	M	Adenocarcinoma	3	I	T2N0M0
**166**	65	M	Adenocarcinoma	3	I	T2N0M0
**167**	39	F	Adenocarcinoma	3	I	T2N0M0
**168**	58	M	Adenocarcinoma	3	II	T2N1M0
**169**	65	M	Adenocarcinoma	3	II	T2N1M0
**170**	54	F	Adenocarcinoma	3	I	T2N0M0
**171**	61	M	Adenocarcinoma	3	I	T1N0M0
**172**	59	M	Adenocarcinoma	3	II	T2N1M0
**173**	42	F	Adenocarcinoma	3	I	T2N0M0
**174**	64	M	Adenocarcinoma	3	I	T2N0M0
**175**	58	M	Adenocarcinoma	3	I	T2N0M0
**176**	62	M	Adenocarcinoma	3	I	T2N0M0
**177**	50	M	Adenocarcinoma	3	I	T2N0M0
**178**	M	57	Adenocarcinoma	-	I	T1N0M0
**179**	62	M	Undifferentiated adenocarcinoma	-	II	T2N1M0
**180**	75	M	Small cell undifferentiated carcinoma	-	I	T2N0M0

**181**	71	M	Small cell undifferentiated carcinoma	-	I	T1N0M0
**182**	68	F	Small cell undifferentiated carcinoma	-	III	T3N1M0
**183**	62	F	Small cell undifferentiated carcinoma	-	III	T4N0M0
**184**	52	M	Small cell undifferentiated carcinoma	-	III	T2N2M0
**185**	65	F	Small cell undifferentiated carcinoma	-	I	T2N0M0
**186**	66	F	Small cell undifferentiated carcinoma	-	II	T2N1M0
**187**	53	M	Small cell undifferentiated carcinoma	-	I	T2N0M0
**188**	56	M	Small cell undifferentiated carcinoma	-	I	T2N0M0
**189**	M	52	Small cell undifferentiated carcinoma	-	I	T2N0M0
**190**	F	51	Small cell undifferentiated carcinoma	-	I	T1N0M0
**191**	M	29	Small cell undifferentiated carcinoma	-	III	T2N2M0
**192**	F	53	Small cell undifferentiated carcinoma	-	I	T2N0M0
**193**	M	54	Small cell undifferentiated carcinoma	-	I	T2N0M0
**194**	M	60	Large cell carcinoma	-	I	T2N0M0
**195**	M	60	Large cell carcinoma	-	II	T2N1M0
**196**	M	67	Large cell carcinoma	-	I	T2N0M0
**197**	F	79	Large cell carcinoma	-	I	T2N0M0

Supplementary Data Table 2: Sequences of primer pairs used in quantitative reverse transcription polymerase chain reaction (RT-qPCR)

	Forward primer/reversed primer
**Oct4**	5'-GTGGAGGAAGCTGACAACAA-3'<br>5'-ATTCTCCAGGTTGCCTCTCA-3'
**Sox2**	5'-GCACATGAACGGCTGGAGCAACG-3'<br>5'-TGCTGCGAGTAGGACATGCTGTAGG-3'
**Nanog**	5'-CCCAAAGGCAAACAACCCACTTCT-3'<br>5'-AGCTGGGTGGAAGAGAACACAGTT-3'
**CXCR4**	5'-CGTCAGTGAGGCAGATGAC-3'<br>5'-TGCAATAGCAGGACAGGATG-3'
**CD133**	5'-TGGATGCAGAACTTGACAACGT-3'<br>5'-ATACCTGCTACGACAGTCGTGGT-3'
**Smo**	5'-TGGTCACTCCCCTTTGTCCTCAC-3'<br>5'-GCACGGTATCGGTAGTTCTTGTAGC-3'
**Bmi-1**	5'-TGGAGAAGGAATGGTCCACTTC-3'<br>5'-GTGAGGAAACTGTGGATGAGGA-3'
**Notch-1**	5'-CCTGAGGGCTTCAAAGTGTC-3'<br>5'-CGGAACTTCTTGGTCTCCAG-3'
**β-catenin**	5'-ACAACTGTTTTGAAAATCCA-3'<br>5'-CGAGTCATTGCATACTGTCC-3'
**Galectin-1**	5'-GCCTGCCCGGGAACAT-3'<br>5'-CTGGCGACCAGACCACAAG-3'
**Galectin-2**	5'-GCTTCAGCGAATCCACCATT-3'<br>5'-GTTCTTGCCCCCAGTTGCT-3'
**Galectin-3**	5'-CCATTTGAAAGTGGGAAACCA-3'<br>5'-CATCATTCACTGCAACCTTGAAG-3'
**Galectin-4**	5'-CCAGCACCTCTTTGACTTTGC-3'<br>5'-CAATGTGTCCACCCTCTGGAA-3'
**Galectin-7**	5'-CTGGCACGGTGCTGAGAAT-3'<br>5'-GGAACCTGCTGGCATTGG-3'

**Galectin-8**	5'-TGAATGCAAATGCCAAAAGC-3'<br>5'-TGGGTTCAAGTGTAGAGCAATATCC-3'
**Galectin-9**	5'-ATGCTGTGGTCCGCAACA-3'<br>5'-GGCAGACTTCGCTCCTCAGA-3'
**ABCG2**	5'-GCAGCTCTTCGGCTTGCA-3'<br>5'-CCCTGTTAATCCGTTCGTTTTT-3'
**ABCB1**	5'-GCTCATCGTTTGTCTACAGTTCGT-3'<br>5'-ACAATGACTCCATCATCGAAACC-3'
**ABCC1**	5'-TGCTGCACCAGTACTTCCACAT-3'<br>5'-CCCCAATGACAGCGGTCTT-3'
**GAPDH**	5'-CAACTACATGGTTTACATGTTC-3'<br>5'-GCCAGTGGACTCCACGAC-3'
